# Enhancing Work Efficiency and Safety Culture in the Food Industry Using Behavioral Patterns: A Video-Based Case Study from Poland

**DOI:** 10.3390/foods15101716

**Published:** 2026-05-13

**Authors:** Patrycja Kabiesz, Grażyna Płaza, Mohammad Gheibi, Małgorzata Żukrowska

**Affiliations:** 1 Department of Production Engineering, Faculty of Organization and Management, Silesian University of Technology, 44-100 Gliwice, Poland; 2 Institute for Nanomaterials, Advanced Technologies and Innovation, Technical University of Liberec, 461 17 Liberec, Czech Republic; 3 Magma Trade Export–Import, 43-440 Goleszów, Poland; m.zukrowska@bielesz.com.pl

**Keywords:** Motion Capture, work efficiency, safety culture, behavioral patterns, Industry 5.0, sustainable development, food industry, meat production

## Abstract

The aim of this study was to examine the impact of professional experience on workflow performance and assess the potential of using behavioral patterns derived from video-based observation to improve work efficiency and safety culture in the food industry. The experiment was conducted at a meat processing plant, with 48 employees divided into four experience groups. Deviations from behavioral patterns, work cycle times, and an efficiency index that considered both speed and accuracy (calculated as a ratio combining task completion time and the average number of deviations) were analyzed. The results showed that experienced employees completed tasks the fastest (8.3 s/cycle) but made the most errors (an average of 4 deviations), while new inexperienced employees worked slower (15.6 s/cycle) but with fewer errors (an average of 1.5 deviations). New employees with previous experience achieved the highest process efficiency (EI = 0.031), demonstrating that a balance between speed and accuracy is crucial. The study is based exclusively on video observation, and no Motion Capture (MoCap) system was used. However, the potential future application of MoCap technology is discussed as a conceptual extension, particularly for enhancing training processes and enabling real-time feedback. Monitoring movements and providing real-time feedback can reduce errors, improve efficiency and support a culture of safety, in line with the principles of Industry 5.0 and sustainability in food production.

## 1. Introduction

Today, enterprises operate in a highly dynamic economic environment where technological innovation is no longer sufficient to determine competitive advantage [1]. Effective human resource management and occupational safety are crucial. In the new era of digital transformation associated with the Industry 4.0 paradigm and the new Industry 5.0 concept, the share of technologies supporting human collaboration with intelligent technical systems is increasing [2,3,4,5]. Industry 5.0 emphasizes the development of human-centric workplaces that take into account employee well-being, cognitive abilities, and physical abilities [4]. Therefore, technologies enabling objective monitoring and analysis of human work processes play a crucial role today [6,7,8].

As part of sustainable economic, social, and environmental development, enterprises must ensure safe and ergonomic working conditions. This is currently defined as one of the fundamental pillars of sustainable industrial systems [9,10]. Furthermore, safety culture is defined as a set of shared values, attitudes, and behaviors directly related to health and safety in the workplace. Safety culture has become a crucial element of responsible enterprise management [10,11]. Modern information technologies allow companies to precisely monitor work processes and identify behavioral attitudes that can impact both work efficiency and occupational safety [12,13].

Among the available approaches, video-based observation methods constitute a practical and non-invasive tool for analyzing human work in real industrial environments. These methods enable the identification of task sequences, behavioral deviations, and execution strategies without requiring complex sensor infrastructure. Compared to advanced motion tracking systems, video analysis is more feasible in operational settings such as food production plants, where technological deployment may be constrained by cost, hygiene requirements, or organizational limitations.

One of the key concepts in this context is behavioral pattern analysis, understood as the identification of repeatable sequences of actions performed during task execution. Deviations from these patterns may indicate inefficiencies, errors, or unsafe practices. Unlike traditional ergonomic assessment methods, which focus primarily on posture and physical load, behavioral analysis emphasizes how tasks are performed, including sequencing, timing, and decision-making routines [14].

Motion Capture (MoCap) technology represents an advanced approach to human motion analysis and is widely discussed in the literature as a tool for capturing detailed kinematic data. MoCap systems record body movements and enable precise analysis of posture, motion, and temporal characteristics of performed tasks. These systems have been successfully applied in ergonomics, rehabilitation, and biomechanics [14,15,16,17,18,19]. However, their implementation in real industrial environments remains limited due to cost, infrastructure requirements, and operational constraints. Therefore, in this study, MoCap is not used as an experimental tool but is considered only as a potential future extension of behavioral analysis systems.

MoCap technologies utilize various types of sensors whose primary purpose is to measure the position, orientation, and movement of body segments. The most commonly used sensors include inertial sensors, optical systems, and wearable technologies. These differ in measurement accuracy, operational limitations, and suitability for industrial applications. A comparison of selected sensors used in MoCap systems is presented in Table 1.

Furthermore, MoCap systems can be categorized by the method of acquiring motion information. The most popular types of tracking systems include optical, inertial, electromagnetic, and mechanical [25,26]. Each of these is characterized by a specific degree of accuracy, mobility, and ease of integration into the industrial work environment. A comparison of the basic types of MoCap systems is presented in Table 2.

Recent years have seen a rapid increase in research interest in MoCap technology. Scientometric analyses indicate a significant increase in the number of publications on movement analysis, ergonomics, and occupational safety since 2015 [10,31,32]. This is related to the rapidly increasing importance of employee health monitoring, predictive safety management, and digital technologies that support the optimization of work processes.

MoCap systems are beginning to be successfully used in ergonomic research, as they analyze joint angles, body movements, and task performance times, allowing researchers to identify risk factors associated with musculoskeletal disorders. Many ergonomic assessment methods, such as RULA, REBA, OWAS, JSI, OCRA, and KIM, have been integrated with MoCap systems to enable automated and objective occupational risk assessment [33,34,35,36,37,38,39]. However, these approaches focus primarily on biomechanical load and posture evaluation rather than on comprehensive behavioral pattern analysis [33,34,35,36,37,38,39].

Optical MoCap systems are considered the most precise technologies for analyzing human movement. These systems utilize multiple cameras to track markers placed on the human body. Although optical systems are accurate, their use is limited to laboratory environments due to cost and difficulty in deploying them on the production floor [40,41].

Despite these advancements, existing studies predominantly address ergonomics, biomechanics, or rehabilitation, while relatively few focus explicitly on behavioral patterns in real industrial task execution. Moreover, studies that do consider behavior often rely on controlled laboratory conditions or advanced sensing technologies, limiting their applicability in real production environments [42,43,44].

A critical gap in the literature concerns the integration of behavioral pattern analysis with performance indicators such as task completion time and error occurrence in real industrial settings. Additionally, the concept of “behavioral deviation” remains insufficiently defined and operationalized in existing studies, particularly in relation to standardized work procedures [33,34,35,36,37,38,39].

The food industry represents a relevant and underexplored context for such analysis due to its high work pace, repetitive tasks, and significant ergonomic and safety risks, as highlighted in prior occupational safety studies, particularly in manual processing and assembly line environments [45]. In this sector, where precise and repeatable movements are essential, analyzing behavioral patterns can significantly influence efficiency, error reduction, and injury prevention.

Therefore, this study aims to address these gaps by applying a video-based behavioral analysis approach in a real industrial environment. The research focuses on identifying deviations from predefined task sequences and analyzing their relationship with task completion time across different levels of employee experience.

Unlike studies centered on motion capture or biomechanical assessment, this work emphasizes observable behavior and task execution strategies derived from video recordings. The proposed approach enables the quantification of efficiency and behavioral variability without the need for advanced motion tracking systems.

The findings contribute to the development of practical methods for process optimization, safety improvement, and strengthening safety culture. Additionally, the results provide a foundation for the future integration of more advanced technologies, such as MoCap, as supportive tools for training and real-time feedback systems in line with Industry 5.0 and sustainable development principles.

## 2. Materials and Methods

### 2.1. Purpose and Scope of the Research

The aim of the study was to determine the impact of professional experience on the way work activities are performed and the potential for using behavioral patterns in the process of shaping work efficiency. The study is based exclusively on video-based observation, and no Motion Capture (MoCap) technology was used at any stage of data collection or analysis.

The scope of the study included:

− identifying behavioral patterns among employees with varying professional experience,
− operationalizing and quantifying deviations from a predefined reference behavioral pattern,
− analyzing the time it takes to complete one work cycle,
− evaluating the relationship between behavioral deviations and work performance indicators.

The study was conducted at a Polish food manufacturing company that produces meat products. The study involved observing actual work activities performed by employees employed as finished product packers.

### 2.2. Characteristics of the Experiment Participants

Forty-eight employees participated in the study, divided into four groups based on their level of professional experience. Each group consisted of 12 individuals, ensuring an even representation of all experience levels in the analysis. Table 3 presents the characteristics of the groups representing different levels of professional experience.

### 2.3. Experiment Stages

The experiment consisted of four main stages:

Stage 1. Standardization of the reference task—all participants underwent training. Employees were presented with occupational health and safety regulations and a video demonstrating the correct procedure for performing the task. This procedure was decomposed into a sequence of discrete actions (task steps), which constituted the reference behavioral pattern used for subsequent analysis.

Stage 2. Work activity recording—each participant performed the same task individually under comparable working conditions. Work execution was recorded using a fixed-position video recording system. No motion tracking sensors or MoCap systems were used.

Stage 3. Behavioral coding and deviation analysis—recorded videos were analyzed using a structured and predefined coding scheme developed on the basis of the reference task model. The reference behavioral pattern was previously decomposed into a sequence of discrete task steps, which served as the benchmark for evaluation. A deviation was operationally defined as any observable departure from this predefined sequence, including deviations in action order, hand positioning, or body movement.

Deviations were classified into three categories: (1) incorrect movement sequence (i.e., performing task steps in an improper order or omitting required actions), (2) incorrect hand positioning (i.e., improper placement or use of hands relative to the task requirements), and (3) excessive or ergonomically inappropriate body movement (e.g., unnecessary bending or leaning beyond what is required for correct task execution). Each identified instance was recorded as a single deviation event, regardless of its duration.

Behavioral coding was conducted by trained evaluators using a standardized coding protocol and a structured observation sheet. To improve accuracy, video recordings were reviewed in slow motion where necessary. To ensure reliability of the coding procedure, a subset of the recordings (20%) was independently analyzed by two researchers. Inter-rater agreement was assessed using Cohen’s kappa coefficient, confirming an acceptable level of consistency in the identification and classification of deviations.

Stage 4. Performance measurement—the time required to complete one full work cycle was measured for each participant based on video timestamps. Additionally, an exploratory Efficiency Index (EI) was calculated as:

(1) $$EI=\frac{1}{T\times (1+E)}$$

where T represents task completion time and E represents the number of observed deviations. The EI is used solely as a relative comparative measure within this study and does not represent a validated performance metric. Higher EI values indicate relatively higher efficiency (shorter time and fewer deviations).

### 2.4. Tools and Methods Used

Employee behavior analysis was conducted solely using video recording and manual motion coding, which allowed for precise observation of postures, body movements, and activities performed in a natural work environment. The recordings enabled the identification of both correct and incorrect task performance patterns, such as incorrect hand positioning, incorrect movement sequences, and excessive body leaning.

A structured observation sheet (coding matrix) was developed prior to analysis, containing predefined task steps and deviation categories. Each recording was analyzed frame-by-frame or in slow motion where necessary to ensure accuracy of coding.

Additionally, the video recordings were used to measure the time taken to complete individual activities and the entire work cycle of each employee. This measurement allowed for the assessment of work efficiency and pace and also enabled comparisons between participants and groups with different levels of professional experience. This approach provided a more comprehensive understanding of both work quality and potential ergonomic risk factors.

### 2.5. Experimental Limitations

The study was designed as a pilot investigation conducted under real-world working conditions, which may have influenced participants’ behavior. In particular, awareness of being observed could have led to the Hawthorne effect, resulting in more cautious or modified task execution. Furthermore, the relatively small sample size and the focus on a single type of work activity limit the generalizability of the findings.

Although inter-rater reliability was assessed, behavioral coding inherently retains a degree of subjectivity, as it depends on the adopted operational definitions and the interpretation of observable actions.

In addition, the study does not include inferential statistical analyses (e.g., hypothesis testing or significance testing), which limits the ability to draw statistically robust conclusions and to compare differences between groups with a high level of confidence. Future research should therefore incorporate appropriate statistical methods to validate the observed relationships and strengthen the reliability of the findings.

It should also be emphasized that no motion capture or sensor-based measurement systems were employed; consequently, the analysis is limited to observable behavioral indicators rather than precise kinematic data.

Moreover, the Efficiency Index proposed in this study should be interpreted as an exploratory measure developed for comparative purposes within the research context, as it has not been externally validated.

Despite these limitations, the results provide a valuable empirical basis for further research on behavioral analysis in real industrial environments and may support the future development and implementation of advanced monitoring technologies, including Motion Capture systems, for improving workplace efficiency and safety.

### 2.6. Diagram of the Research Process

The research process can be described as follows:

1. Identification of production processes within the company.
2. Detailed analysis of the selected production process.
3. Selection of the workstation where the experiment was conducted.
4. Preparation of the workstation and on-the-job training.
5. Training in occupational health and safety regulations.
6. Employee performance of the task with video recording.
7. Analysis of deviations from the behavioral pattern.
8. Measurement of efficiency (cycle time).

### 2.7. Research Model

**Figure 1:**
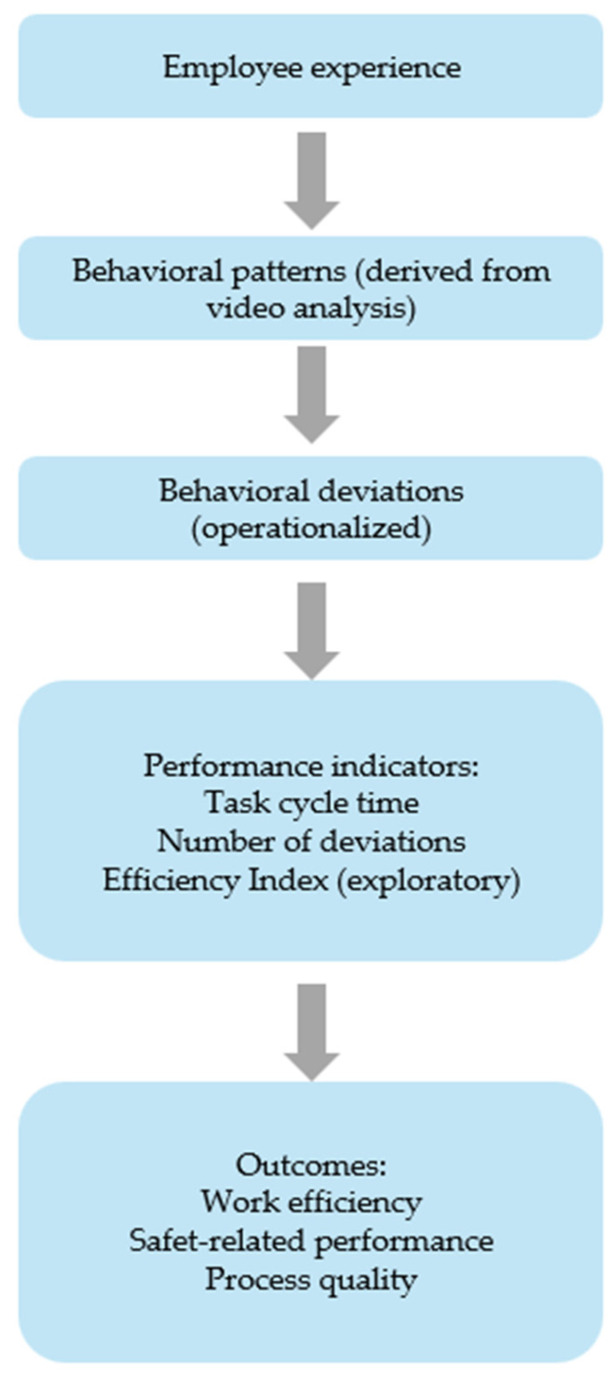
Research model of work efficiency and safety.

Based on the purpose and scope of the research, a research model was developed (Figure 1), which illustrates the relationships between employees’ professional experience, video-derived behavioral patterns, and performance outcomes in the context of work efficiency and safety.

The model assumes that the level of professional experience influences the way tasks are performed, which is reflected in the formation of behavioral patterns. These patterns are identified through structured video analysis and further examined by means of operationally defined behavioral deviations. The number and type of deviations, together with task completion time, directly affect performance indicators, including work efficiency and task execution quality.

In contrast to sensor-based approaches, the proposed model is based exclusively on video-based behavioral observation and does not include Motion Capture (MoCap).

The model links behavioral analysis with key outcomes such as work efficiency, safety-related performance, and process quality. In particular, the combined analysis of task cycle time and deviation frequency enables the assessment of trade-offs between speed and accuracy in task execution.

Motion Capture (MoCap) systems are not included in the core model and are considered only as a potential future extension. Such systems could support automated movement tracking and real-time feedback, facilitating the identification and correction of behavioral deviations during task execution.

This approach provides a basis for designing targeted interventions aimed at improving both work performance and working conditions while remaining applicable in real industrial environments without the need for advanced motion tracking technologies.

## 3. Results

### 3.1. Characteristics of the Research Company

The research was conducted at a Polish food company focused on pork processing, carcass cutting, and the production and distribution of cured meat products. The plant currently offers approximately 150 product ranges, spanning five basic categories: finely, medium and coarsely ground products, block products, and smoked meats.

The company has been operating continuously since 1945, and in 2005, a modern 4000 m^2^ production facility was opened, employing approximately 140 people. Since 2018, the plant has been conducting systematic observational research, including video recording and photographic documentation of staff work. The analyses focus primarily on the production process of medium-ground sausage (hereinafter referred to as “sausage”).

The sausage-making process involves a total of 11 stages, including six primary processes: cutting, curing, production, smoking, cooling, and packaging, and five auxiliary processes: storage, cleaning, internal transport, quality control, and preparation of raw materials for further processing. Production begins with the receipt of raw materials, their temperature and weight control, and then transport to the cold store and cutting room. Key technological operations include dry curing, meat grinding, mixing ingredients, shaping and stuffing the filling, smoking, heat treatment, cooling, and modified atmosphere packaging (MAP), followed by order picking and product preparation for shipment.

**Figure 2:**
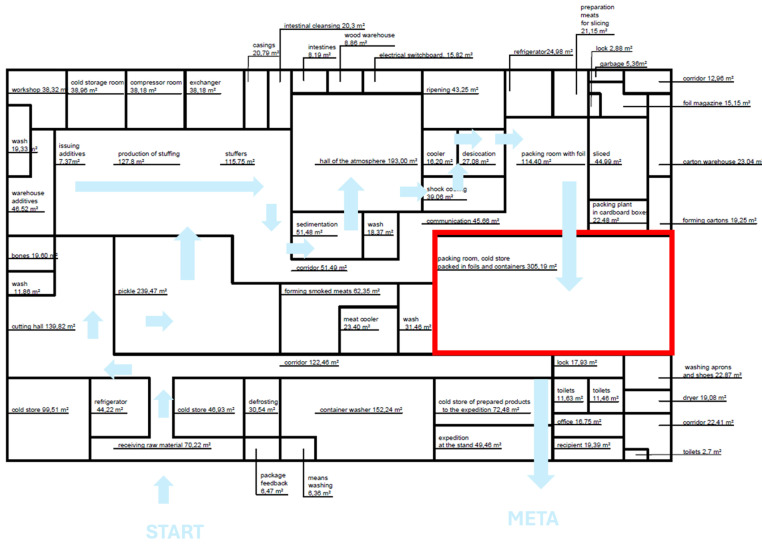
A layout of the facility, including the flow of raw materials and products during sausage production.

Figure 2 presents the functional layout of the plant, along with the floor area of individual rooms and the flow direction of raw materials and products during the production process. The area covered by the research experiment is highlighted in red and includes the packaging room and the finished product cold store. The selected zone is characterized by a high intensity of manual work and a significant share of transport and manipulation operations, which justifies its particular importance in ergonomic analysis and assessment of the efficiency of the production process.

### 3.2. Description of the Production of Medium-Ground Sausages

The technological process begins with the receipt of raw material delivered by external suppliers. The employee responsible for receiving the material is responsible for checking the temperature printout from the transport trailer, measuring the temperature of the material, and weighing it. Pork sides are delivered on hook-lift trailers and then transported to the cold store using a rail system with rolling discs. Proper placement of the sides is crucial for storage—they must not touch each other, as pressure points can negatively impact the sensory quality of the meat.

The next step is the cutting room, where the sides are cut into primary parts using cutting saws, and then into culinary cuts using knives. The resulting parts are classified by employees and placed in appropriately labeled containers.

A key process in meat processing is curing, the purpose of which is to impart appropriate flavor and aroma characteristics, extend the product’s shelf life, develop the proper texture, and preserve the meat’s natural color. Before this stage begins, the raw material is weighed. There are three basic curing methods: dry (involving rubbing the meat with a curing mixture), wet (based on the application of a curing solution through soaking or injection), and a combined method combining both approaches. For medium-ground sausages, dry curing is used, during which the meat in loading trolleys is evenly coated with the curing mixture and then reweighed.

The raw material is then ground in a cutter. Depending on the required particle size, knives with varying numbers of blades and meshes with a specific mesh diameter are used. The sharpness of the knives is crucial—improperly maintained knives can lead to crushing the meat and excessive heating, which reduces the quality of the final product. Water or ice is added during the cutting process to achieve a homogeneous mass with a temperature no higher than 12 °C. To ensure even distribution of ingredients and achieve the desired consistency, the stuffing is mixed in a mixer.

The next step is shaping the stuffing, giving it a specific shape, depending on the type of casing used—natural or artificial. This process is carried out using a stuffer. The formed products are hung on smoking sticks, placed on trolleys, and sent to a settling room, where color stabilization and flavor evenness occur for approximately two hours at a temperature of approximately 30 °C.

Smoking and heat treatment are essential stages in sausage production, influencing the development of characteristic sensory characteristics, color retention, and limiting undesirable microbiological processes. These processes are carried out in Atmos smoking and steaming chambers and include drying, smoking, steaming, and water cooling.

After heat treatment, the products undergo wet and dry cooling. After reaching a temperature of approximately 5 °C, they are sent to the packaging stage. Modified atmosphere packaging (MAP) is commonly used in the meat industry, which involves creating the appropriate gas composition (oxygen, carbon dioxide, nitrogen) inside the packaging, which extends the product’s shelf life.

The final stage of the process is the completion of shipment batches. The products are labeled, packed in cartons according to customer requirements, and then transported by pallet trucks to the finished product refrigeration facility, where they await shipment.

**Figure 3:**
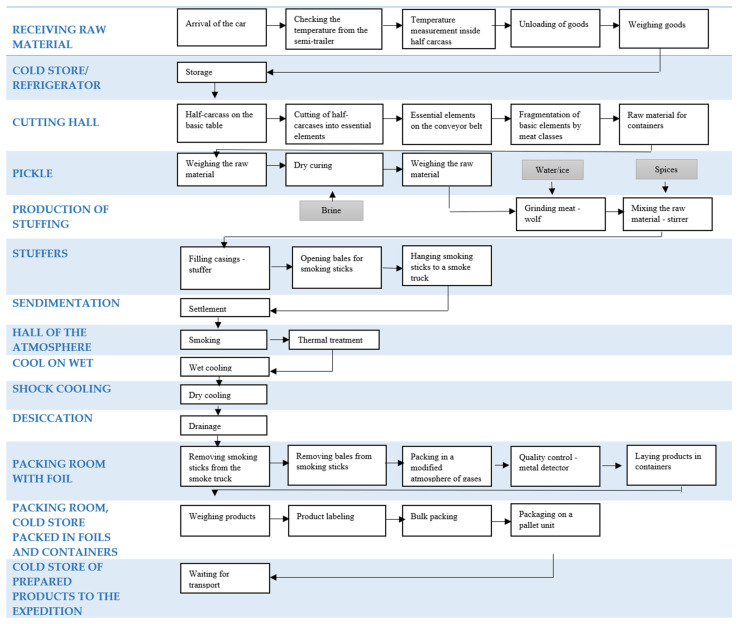
The production process of medium-ground sausage.

Figure 3 shows a simplified, comprehensive sausage production process, including a list of rooms and the individual activities performed within them.

### 3.3. Measurement Results—Cycle Time and Number of Deviations from the Pattern

**Figure 4:**
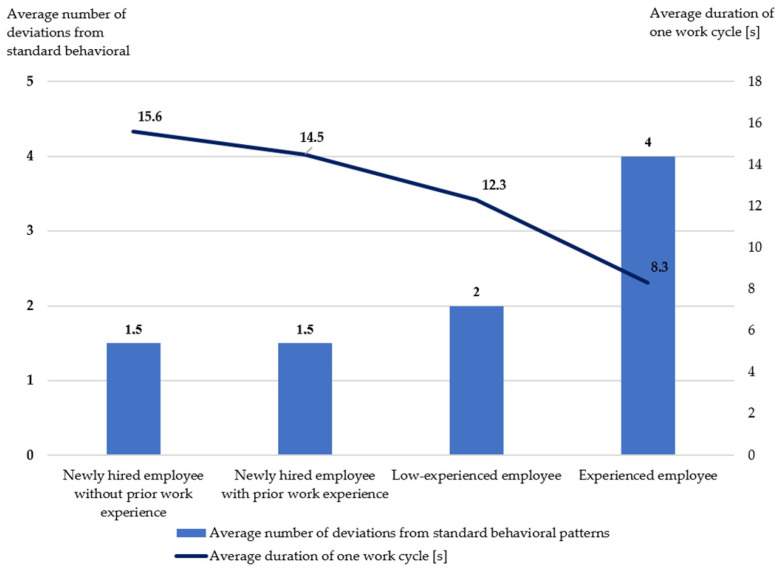
Average cycle time and number of deviations from the standard behavioral pattern.

Based on video recordings of the finished goods packer’s work station, the number of deviations from the behavioral pattern and the time it took to complete one work cycle for each participant were analyzed. Figure 4 presents the average values of cycle time and number of deviations across different employee experience levels.

The analysis showed that employees with the highest level of experience completed the task the fastest (average cycle time—8.3 s) but also made the most errors (average number of deviations from the behavioral pattern—4). In contrast, the newly hired employee with no professional experience took the longest to complete the task (average cycle time—15.6 s), and the fewest errors were made (average number of deviations from the behavioral pattern—1.5).

The presented results suggest that the time it takes to complete a work activity decreases with increasing professional experience. Simultaneously, the number of deviations from the behavioral pattern increases, which may suggest a stronger influence of habitual and automated behavior among more experienced employees. However, these observations should be interpreted as correlations rather than causal relationships, as no statistical inference was applied.

These results indicate a trade-off between task execution speed and adherence to the predefined behavioral pattern. With increasing professional experience, task execution becomes faster but also more prone to deviations.

It should be emphasized that the presented results are descriptive in nature, and no statistical inference was performed; therefore, the observed differences should be interpreted as indicative trends rather than statistically confirmed relationships.

Furthermore, the experiment shows that experienced employees have established, often automated, patterns of action. A habit can be defined as learned, unconscious behavior that manifests itself in specific situations (procedural memory) [46,47]. Considering research findings [48,49], which indicate difficulties in modifying established behavior patterns, it was assumed that an experienced employee who performs tasks based on unconscious memory may need more time to change their workflow.

In contrast, a newly hired employee, relying primarily on conscious memory, requires more time to complete a task because their actions are based on analysis and control of each step of the process. Their behavior is not yet automatic, but rather deliberate and sequential.

This phenomenon can be explained through two models of employee functioning. The first is the conscious model (based on working memory), characteristic of new employees who perform tasks more slowly, with high concentration and control over individual movements. The second is the automatic model (based on procedural memory), typical of experienced employees who perform tasks routinely and at a faster pace but often omit certain details, including safety aspects. Research indicates that over time and with experience, working memory is gradually replaced by procedural memory, leading to the automation of behavior. This process contributes to increased work speed, but it can also facilitate routine-based errors and reduce compliance with established patterns of behavior.

### 3.4. The Relationship Between Experience and Work Efficiency

The results confirm the existence of a nonlinear relationship between professional experience and efficiency. Employees with more experience perform tasks faster but with less accuracy. As a result, overall process efficiency may be reduced due to errors that require subsequent correction or generate the risk of accidents.

The EI is used exclusively as a relative comparative measure within this study and does not represent a validated performance metric.

The highest EI value (0.031) was observed among newly hired employees with prior work experience, indicating a relatively favorable balance between speed and accuracy. Lower EI values were observed in more experienced groups, where faster execution was accompanied by a higher number of deviations.

These findings suggest that work efficiency does not increase linearly with experience and that excessive task automation may lead to reduced compliance with the predefined behavioral standard.

This indicator decreases with increasing time and error frequency; higher EI values indicate greater overall efficiency. Table 4 summarizes the effects of employee experience on task completion time, error rate, and workflow efficiency.

The Efficiency Index (EI), which takes into account both work time and the number of errors, best reflects overall productivity. The highest EI value (0.031) is achieved by the group of new employees with previous experience. This means they work relatively quickly and make few errors.

The lowest EI values (0.024–0.025) are observed among more experienced employees—despite shorter work time, a higher number of errors reduces their overall productivity.

The results indicate that efficiency does not increase linearly with professional experience. However, no statistical significance testing was performed; therefore, differences between groups should not be interpreted as statistically confirmed.

The most favorable EI level is observed among employees with moderate experience, where a balance is maintained between task completion speed and accuracy. In this group, completion time is relatively short, and the number of errors remains low, which translates into the highest EI values. In the case of highly experienced employees, however, excessive routine and automation of tasks can lead to a decrease in accuracy. As a result, the increasing number of errors reduces the overall efficiency of the process, despite the shortened completion time. Ultimately, it can be concluded that speed alone is not a sufficient indicator of high efficiency. Maintaining a balance between the pace of work and the quality of the work performed is crucial.

### 3.5. Conceptual Implications for the Use of Motion Capture Systems

The results obtained in this study provide a basis for considering the potential application of Motion Capture (MoCap) systems as a supportive tool for behavioral analysis and training. It should be clearly emphasized that no MoCap system was implemented or tested in the present study, and the following considerations are purely conceptual.

Rather than referring to a specific commercial solution (e.g., MCU MOVE), MoCap technology is discussed here in general terms as a class of motion tracking systems described in the literature. Such systems are based on tracking the position of body segments using markers or markerless vision-based approaches, enabling the analysis of movement patterns, posture, and task execution dynamics [50].

In contrast to the video-based method applied in this study, MoCap systems could potentially provide more detailed and continuous kinematic data, including displacement, velocity, and acceleration of body segments. This type of data could support the automatic detection of deviations from predefined behavioral patterns and facilitate more objective performance assessment.

**Figure 5:**
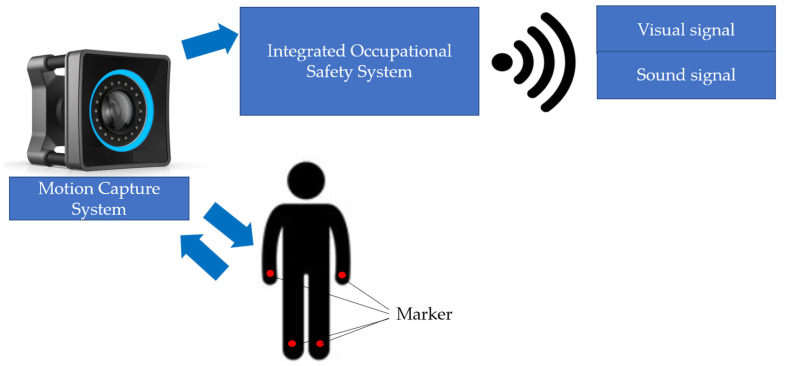
Method of using the MoCap system.

Figure 5 schematically illustrates how the MoCap system can be used to create optimal working conditions.

**Figure 6:**
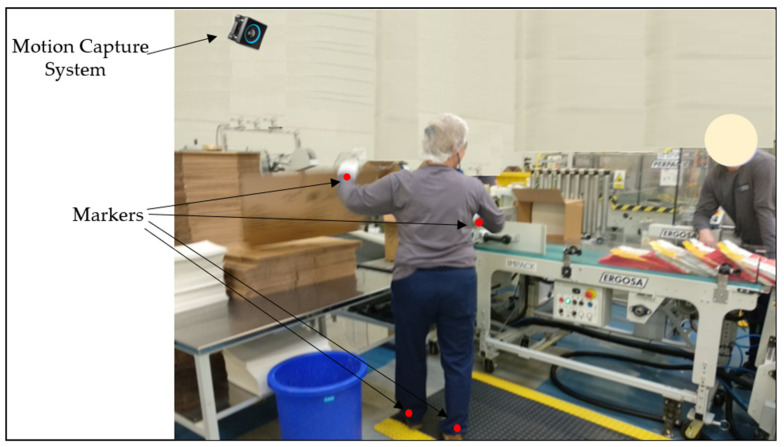
Proposal for using the MoCap system in the analyzed company.

Additionally, Figure 6 presents a proposed location for the MoCap system at the analyzed workstation within the company. It was suggested to use one camera and four markers placed on the legs and arms. This will minimize the costs of implementing the proposed solution, and the installation process will be straightforward.

**Figure 7:**
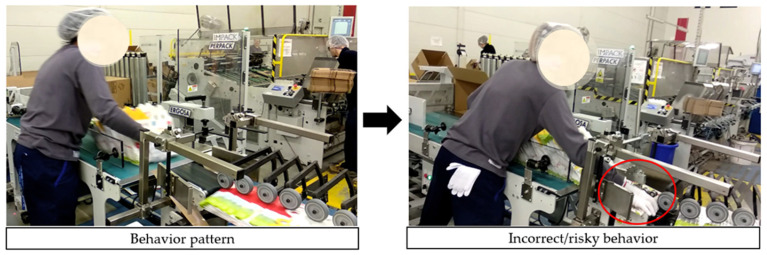
Use of the MoCap system in the company.

Figure 7 shows an employee’s inappropriate behavior (deviation from the safety behavior pattern). The employee placed his hand in a moving machine part. The location of the MoCap system sensor in a prohibited zone triggered an audible warning, enabling a quick response and avoidance of an accident.

It is worth noting that informing an employee of the intention to conduct an observation may require extra care to perform their work properly, in accordance with safety regulations (if the person being observed is aware of them). After the observation ends, the motivation to perform their work safely may decrease. Unnatural, yet appropriate, employee behavior occurs when they know they are being observed, and management can impose consequences for non-compliance with safety regulations. In the absence of external supervision, there is no motivation for safe behavior, and therefore, the employee does not identify with the company’s regulations regarding occupational safety and develop safe behavior habits. In everyday life, this type of behavior is very common in society, for example, among drivers who, upon seeing a police car, slow down and fasten their seat belts, then accelerate and unfasten them. Developing safe habits among employees is a long-term process that can be supported by the MoCap system. The potential benefits of implementing this system in a company include:

− increased work process efficiency,
− reduced number of accidents at work,
− improved quality of finished products,
− providing feedback to those observed and initiating thought-provoking conversations about safety,
− assistance in setting goals and actions to improve safety,
− shaping a safety culture (observing employee behavior is one measure of the level of safety culture development),
− identifying problems that occur in a given area but have not yet been defined.

In summary, the MoCap system uses an observational method to analyze human behavior. Its key feature, distinguishing it from other systems, is the collection of information about employee behavior in their natural settings throughout the entire work shift. This system can be used in a company temporarily, during the training of a newly hired employee until the desired parameters are achieved, or as a control system for employees with professional experience, and when necessary, it is used to correct inappropriate behavior.

## 4. Discussion

Based on the results of the conducted research, together with insights from the international literature on MoCap and related motion analysis systems, it can be concluded that these technologies are discussed in the literature as potentially supportive in shaping safety cultures in industrial companies. However, it should be clearly emphasized that the present study does not provide empirical evidence for these effects, and therefore MoCap should be interpreted here as a conceptual and literature-based consideration rather than a validated intervention. Compared to traditional approaches to safety culture, which emphasize periodic employee training and direct supervision, MoCap-based systems are described in the literature as enabling continuous monitoring of employee behavior within the work process [51,52,53].

The application of MoCap in a company, as presented in this study, is discussed in conceptual terms rather than experimentally implemented, which limits the possibility of drawing causal conclusions regarding its effectiveness. Accordingly, MoCap systems are considered here as a theoretical concept that may potentially contribute to shaping safety culture through several mechanisms. First, MoCap enables the monitoring of employee behavior within the actual work process. In principle, such monitoring may allow for the detection of abnormal behaviors or movements that could lead to injury. Nevertheless, evidence for its real-world effectiveness in complex industrial environments remains limited. Research indicates that MoCap systems may support musculoskeletal disorder (MDS) prevention and improve workplace ergonomics [54,55], although these findings are primarily derived from controlled or pilot studies.

Another important aspect is the ability to identify deviations from accepted work patterns without the need for constant supervision by supervisors. As emphasized by authors specializing in the digitalization of industrial processes, automating employee behavior monitoring helps reduce the subjectivity of assessments and increases the consistency of implemented safety standards [56,57,58,59,60,61,62,63,64,65]. In this context, MoCap is described as a potential enabler of data-driven decision-making, in which decisions are based on behavioral indicators, although such applications remain largely at a conceptual or early implementation stage.

Another significant element of MoCap’s impact on safety culture is the provision of immediate feedback. Feedback mechanisms, both auditory and visual, are reported in the literature to potentially improve workers’ behavioral awareness and support safer task execution. According to learning theories and research findings on organizational behavior, rapid and direct feedback promotes more effective consolidation of desired patterns and reduction in risky behaviors [66,67,68]. As a result, employees may gradually internalize appropriate practices, which is considered in the literature as a foundation of a sustainable safety culture. However, long-term evidence from industrial-scale deployments is still scarce.

Compared to traditional training methods, which are episodic and sometimes theoretical in nature, MoCap is described as enabling continuous monitoring and dynamic adjustment of employee behavior during work. This approach may support a more individualized learning process, consistent with the concept of situational learning. As various studies indicate, personalizing ergonomic interventions and leveraging real-world data may improve the effectiveness of preventive measures [69,70,71,72], although these conclusions should be interpreted cautiously due to variability in study contexts and methodologies.

MoCap technology also finds a place within the framework of Industry 4.0 and the evolving concept of Industry 5.0, which places particular emphasis on the importance of integrating humans with intelligent technical systems. Integrating employee behavior data with production management systems (MES, ERP) is proposed in the literature as a means to support a comprehensive approach to managing safety, efficiency, and quality of the production process [73,74,75,76]. At this stage, however, such integration remains largely conceptual and dependent on technological and organizational readiness.

The literature also highlights the growing importance of motion monitoring technology within predictive safety management. Analysis of data collected by such systems may enable the identification of behavioral patterns leading to accidents before they occur, which represents an important step toward proactive risk management [77,78,79,80,81,82,83,84,85]. Nevertheless, robust empirical validation of such predictive capabilities in real industrial environments is still limited.

It is also important to acknowledge several challenges associated with MoCap implementation in industrial environments, including high implementation costs, employee acceptance, data privacy concerns, and integration with existing production management systems. These factors may significantly limit practical applicability and should be carefully addressed in future empirical research.

In summary, MoCap should be understood primarily as an emerging and promising conceptual approach rather than an empirically validated solution within the scope of this study. Its potential relevance lies in supporting a transition toward more data-informed and proactive safety management approaches; however, the current evidence base is not sufficient to confirm its direct impact on safety culture in industrial settings. Integrating motion analysis technologies with organizational processes remains an area for further empirical investigation.

## 5. Conclusions

A review of the conducted study and existing literature highlights the growing interest in motion analysis technologies in the context of work efficiency and occupational safety. However, it should be explicitly stated that MoCap technology was not used in the empirical part of this study, and therefore all references to MoCap represent a theoretical and literature-based discussion rather than an experimentally implemented method. The empirical analysis was based exclusively on video observation and behavioral coding of recorded work activities.

Key findings include:

− The study confirms that professional experience influences both the pace and quality of work—more experienced employees complete tasks faster but make more errors, which may reduce their overall efficiency. Employees with moderate experience achieve the most balanced performance results.
− Automation of routine activities (procedural memory) can increase the risk of errors, indicating that speed alone is not a sufficient indicator of efficiency. The balance between pace and adherence to a standard is crucial.
− The results demonstrate a relationship (not causation) between experience level, task execution time, and behavioral deviations, highlighting the importance of behavioral pattern analysis in industrial environments. It should be noted that “deviation” in this study refers specifically to observable differences in task execution recorded through video coding, rather than technologically measured motion tracking outputs.
− Based on literature analysis, MoCap systems are described as tools that may enable precise movement tracking and assessment of deviations from desired patterns; however, these capabilities were not tested or validated in the present empirical study.
− In this study, however, MoCap is proposed as a potential tool rather than experimentally validated, particularly in the context of employee training, behavior correction, and safety culture development.

Despite these contributions, the study has several limitations. First, the research was conducted as a pilot study with a relatively small sample size (48 participants), which limits the generalizability of the results. Second, the analysis was limited to a single type of work activity in one company, which may not reflect other industrial contexts. Third, the study does not include inferential statistical analysis (e.g., hypothesis testing or significance analysis), which restricts the ability to confirm statistical differences between groups. Additionally, behavioral coding procedures require further clarification and validation, including inter-rater reliability assessment, which was not included in the present study. Finally, constructs such as the Efficiency Index and “deviation” should be interpreted cautiously, as they lack external validation and established theoretical grounding.

Future research should address these limitations by:

− incorporating larger and more diverse samples across different industrial sectors,
− applying inferential statistical methods to validate observed relationships,
− experimentally implementing MoCap systems to verify their effectiveness in real-time behavioral monitoring and feedback,
− and further developing the concept of behavioral patterns as measurable and operational constructs in industrial environments.

In summary, this study contributes to the literature by providing empirical evidence on the relationship between professional experience and behavioral performance indicators in a real industrial setting based on video observation. While the results highlight the importance of balancing speed and accuracy, the role of MoCap technology should be understood exclusively as a conceptual and future research direction and not as part of the empirical findings of this study.

## Figures and Tables

**Table 1:** Comparison of sensors used in MoCap systems.

Sensor Type	Operating Principle	Advantages	Limitations	Sources
Accelerometer	Measures linear acceleration of body segments in three axes	Simple structure, low cost, ease of integration in wearable systems	Cannot independently determine orientation without sensor fusion	[14,20,21]
Gyroscope	Measures angular velocity around one or more axes	High sensitivity to rotational motion, enables precise dynamic analysis	Susceptible to drift errors over time, requires calibration	[14,20,21]
Magnetometer	Measures orientation relative to the Earth’s magnetic field	Improves orientation estimation when combined with other sensors	Sensitive to electromagnetic interference in industrial environments	[14,20,21]
Optical camera	Tracks markers or body silhouette using vision-based systems	Very high spatial accuracy and detailed motion reconstruction	Requires controlled lighting conditions and limited occlusions	[21,22,23]
Wearable Flexible Sensors	Measure deformation of flexible materials integrated into garments	High user comfort, suitable for continuous and long-term monitoring	Lower measurement precision compared to optical systems	[23,24]

**Table 2:** Comparison of MoCap systems.

System Type	Measurement Principle	Advantages	Limitations	Sources
Optical (Marker-Based)	Cameras track reflective markers placed on predefined anatomical points	Very high spatial accuracy and precise motion reconstruction	High cost, complex setup, and limited mobility in industrial environments	[27]
Optical (Markerless)	Computer vision and AI algorithms estimate body posture without physical markers	Natural movement, high user comfort, and non-invasive measurement	Sensitivity to lighting conditions, occlusions, and reduced accuracy compared to marker-based systems	[28]
Inertial (IMU-Based)	Wearable inertial sensors measure linear acceleration and angular velocity	High mobility, portability, and suitability for real-world industrial applications	Sensor drift, need for calibration, and lower positional accuracy	[29,30]
Electromagnetic	Sensors detect position and orientation based on electromagnetic field variations	Accurate measurements in controlled environments without line-of-sight requirements	Highly sensitive to electromagnetic interference and environmental disturbances	[27]
Mechanical	Exoskeleton-based systems measure joint angles through mechanical linkages	High precision in joint angle measurement and repeatability	Restricts natural movement and may affect task performance	[27]

**Table 3:** Characteristics of groups representing different levels of professional experience.

Group	Employment Duration in the Company	Total Work Experience
Newly hired employee without prior work experience	up to 3 months	first job
Newly hired employee with prior work experience	up to 3 months	more than 12 months
Low-experienced employee	3–12 months	up to 2 years
Experienced employee	more than 12 months	more than 5 years

**Table 4:** The relationship between the level of professional experience and the efficiency of the work process.

Group	Time [s]	Number of Errors	Efficiency Index
Newly hired employee without prior work experience	15.6	1.5	0.026
Newly hired employee with prior work experience	12.8	1.5	0.031
Low-experienced employee	10.1	3	0.025
Experienced employee	8.3	4	0.024

## Data Availability

The original contributions presented in this study are included in the article. Further inquiries can be directed to the corresponding authors.

## Abbreviations

The following abbreviations are used in this manuscript:

MoCap	Motion Capture
